# A252 DO GLIM CRITERIA FOR DIAGNOSING MALNUTRITION AGREE WITH SGA IN HOSPITALIZED PATIENTS RECEIVING TPN?

**DOI:** 10.1093/jcag/gwab049.251

**Published:** 2022-02-21

**Authors:** E Vantomme, B Sulymka, M Stevens, D Duerksen

**Affiliations:** University of Manitoba, Winnipeg, MB, Canada

## Abstract

**Background:**

The Global Leadership Initiative on Malnutrition (GLIM) proposed a new two-step model for diagnosing malnutrition in 2019. A combination of two etiologic and three phenotypic criteria are used to assess malnutrition. The Subjective Global Assessment (SGA) is the most well validated assessment tool for diagnosing malnutrition in hospitalized patients. Evaluation of the performance of GLIM criteria in comparison to SGA is necessary before implementing this new diagnostic tool in practice.

**Aims:**

To compare GLIM criteria to SGA in assessing malnutrition severity in hospitalized patients requiring parenteral nutrition (PN).

**Methods:**

This is a retrospective analysis of a prospectively collected database of malnourished hospitalized adult patients requiring PN admitted between March 2020 and March 2021 to an academic hospital in Winnipeg, Canada. 172 cases were evaluated. GLIM malnutrition screening was considered positive if one etiologic (high CRP or low food intake) and one phenotypic (weight loss or low BMI) criteria were identified. Sensitivity, specificity, positive predictive value (PPV), and negative predictive value (NPV) were calculated and expressed with a Wilson 95% confidence interval.

**Results:**

The prevalence of malnutrition using SGA B or C criteria was 82.3% (CI 73.8, 85.5). Using GLIM criteria, the prevalence of malnutrition was 33.7% (27.1, 41.1). The prevalence of severe malnutrition using SGA C was 32.6% (26.0, 40.0) and using GLIM criteria the prevalence was 19.2% (14.0, 25.7). Using any combination of GLIM criteria versus SGA B or C combined, the PPV was 100% (90.4, 100) and the specificity was 100% (89.9, 100); NPV was 30.0% (22.0, 38.5) and the sensitivity was 42.0% (34.1, 50.4). Using any combination of GLIM criteria versus only SGA C patients, PPV decreased to 72.4% (59.8, 82.3) and specificity was 86.2% (78.8, 91.3); NPV increased to 87.7% (80.4, 92.5) and sensitivity was 75.0% (62.3, 84.5). Comparing severely malnourished patients by GLIM criteria to only SGA C patients, PPV was 97% (84.7, 99.5) and specificity was 99.1% (95.3, 100). NPV was 82.7% (75.6, 88.1) and sensitivity was 57.1% (44.1, 69.2).

**Conclusions:**

Using SGA as the gold standard for diagnosing malnutrition in hospitalized patients requiring PN, GLIM criteria had a very high PPV but unacceptably low NPV in diagnosing malnutrition. The NPV improved when GLIM criteria was compared only to severely malnourished patients by SGA. Importantly, in comparing severely malnourished patients by GLIM and SGA criteria, the sensitivity was also unacceptably low. Based on these results, GLIM criteria are most useful in confirming the diagnosis of malnutrition or severe malnutrition; a negative result should not reassure clinicians that severe malnutrition is absent. Further studies evaluating GLIM criteria are needed before it replaces SGA as a decision making tool.

**Funding Agencies:**

None

